# Alveolar soft part sarcoma in childhood and adolescence: Report of three cases and review of literature

**DOI:** 10.3389/fped.2022.937112

**Published:** 2022-11-18

**Authors:** Yudi Zhang, Ying Wang, Hao Wang, Chuan Wen, Xiaochuan Wu

**Affiliations:** ^1^ Department of Pediatrics, Second Xiangya Hospital, Central South University, Changsha, China; ^2^ School of Instrument and Electronics, North University of China, Taiyuan, China

**Keywords:** alveolar soft part sarcoma, children, TFE3, pathology, treatment

## Abstract

Alveolar soft part sarcoma (ASPS) is a rare soft tissue sarcoma with unique tumor characteristics, which is rare in children. Herein, we present the immunophenotype, treatment, and prognosis of three children with ASPS from The Second Xiangya Hospital of Central South University, and 51 children with ASPS have been reported in the previous literature, along with a focused review of the clinical features, pathological features, differential diagnosis, treatment, and prognosis of ASPS in pediatric patients.

## Introduction

Alveolar soft part sarcoma (ASPS) is a rare soft tissue sarcoma reported by Christopher et al. in 1952 (1). It has the characteristics of unknown tissue source, high invasiveness, easy metastasis, and poor prognosis. It accounts for less than 1% of adult soft tissue sarcoma and 1%–2% of childhood soft tissue sarcoma. It is most common in female patients with a median age of 20 years old and is relatively rare in children (2). It has been reported that ASPS in children has lower aggressive behavior and different biological characteristics, and the prognosis is better than that in adults (3). Therefore, we collected the clinical data of three children with ASPS from The Second Xiangya Hospital of Central South University, reviewed and discussed the literature on the immunophenotype, treatment, and prognosis of 51 children, which have been reported in the previous literature (Table 1). To further understand the clinical features, pathological features, differential diagnosis, treatment, and prognosis of the tumor in children.

**Table 1:** Literature review of child ASPS.

No	Authors	Years	Age (years)	Sex	Location	Tumor size (cm)	Metastasis	Molecular confirmation	Treatment	Follow-up	References
1	Chenchen Liu	2017	17	M	Lung	16.0	None	Y	SR	Alive at 16 months	(4)
2	Liping Che	2009	4	M	Tongue	N/R	None	N/R	ACT + RT + SR	N/R	(5)
3	Fengxia Yuan	2018	9	M	Intracerebral	3.0	None	Y	SR	Alive at 12 months	(6)
4	Chan	2004	4	M	Orbital	N/R	None	N/R	SR	Alive at 24 months	(7)
5	Penticuff	2019	9	F	Bladder	4.2	None	Y	SR	Alive at 7 months	(8)
6	Sood	2018	8	F	Shoulder	N/R	None	Y	SR	Alive at 3 months	(9)
7	Marchac	2007	2	F	Tongue	4.0	Lung	Y	ACT + SR	Alive at 52 months	(10)
8	Asano	2019	11	F	Cheek	N/R	Lung, breast, head	Y	ACT + Pazopanib + SR	N/R	(11)
9	Qiu	2017	3	M	Penis	2.1	None	Y	SR + ACT	Alive at 28 months	(12)
10	Jia	2011	2	F	Abdominal wall	2.5	None	N/R	SR	Alive at 6 months	(13)
11	Ata	2014	9	M	Thigh	10.0	None	N/R	SR	Alive at 48 months	(14)
12	Nava-Castaneda	2017	4	F	Orbital	3.5	None	N/R	SR	Alive at 6 months	(15)
13	Shires	2011	2	M	Thyroid gland	4.5	Lung	N/R	SR	Died	(16)
14	Bassichis	2003	9	M	Tongue	N/R	None	N/R	SR	N/R	(17)
15	Suarez	2020	13	F	Tongue	2.6	None	Y	SR + ACT	N/R	(18)
16	Yoshida	2000	2	F	Tongue	2.0	None	N/R	SR + ACT	Alive at 86 months	(19)
17	Baglam	2009	18	F	Tongue	6.0	Lung	N/R	SR + RT + ACT	Died	(20)
18	de Fátima Correia-Silva	2006	17	F	Tongue	2.0	None	N/R	SR	Alive at 12 months	(21)
19	Lee	2014	17	F	Uterine Cervix	1.6	None	Y	SR	Alive at 24 months	(22)
20	Chakrabarti	2014	14	M	Tongue	4.0	None	N/R	SR	N/R	(23)
21	Usawachintachit	2016	18	M	Bladder	1.7	None	Y	SR	Alive at 28 months	(24)
22	Yilmaz	2004	10	F	Glabella	0.5	None	N/R	SR	Alive at 12 months	(25)
23	Van Buren	2009	13	F	Breast	2.5	Lung	N/R	N/R	N/R	(26)
24	Yigitbasi	2004	14	M	Paranasal sinus	N/R	None	N/R	SR + RT	Alive at 24 months	(27)
25	Asokan	2017	18	F	Forearm	7.0	Lung	N/R	SR + ACT	Alive at 11 months	(28)
26	Noussios	2010	3	M	Tongue	3.3	None	N/R	SR	Alive at 42 months	(29)
27	Dutta	2021	13	F	Paranasal sinus	N/R	None	Y	SR + ACT + RT	Alive at 15 months	(30)
28	Dutta	2021	4	F	Tongue	1.2	None	Y	SR + RT	Alive at 13 months	(30)
29	Ruffle	2019	0.92	F	Tongue	1.6	None	Y	SR	Alive at 14 months	(31)
30	Kumar	2010	7	M	Tongue	2.5	None	N/R	SR + ACT	Alive at 11 months	(32)
31	Kos	2011	17	M	Buttocks	N/R	Lung, head	Y	SR + ACT + RT	Metastasis after 24 months	(33)
32	Hanzer	2010	14	F	Thigh	4.0	Lung	N/R	SR + ACT + RT + Sunitinib	Alive at 2 months	(34)
33	Argyris	2013	4	M	Tongue	1.6	None	Y	SR	Alive at 7 months	(35)
34	Argyris	2013	13	M	Cheek	3.2	None	Y	SR + ACT	Alive at 12 months	(35)
35	Tao	2016	13	M	Intracerebral	N/R	None	Y	SR + RT	Recurrence after 24 months	(36)
36	Caporalini	2021	16	F	Intracerebral	N/R	None	Y	SR + RT	Alive at 4 months	(37)
37	Emmez	2015	11	F	Intracerebral	N/R	None	Y	SR + RT + ACT (first treatment) SR + ACT (second treatment)	Recurrence after 45 months	(38)
38	Aggarwal	2021	16	M	Thyroid Gland	5.0	Lung	Y	SR	N/R	(39)
39	Tanabe	2018	9	F	Bladder	1.0	None	Y	SR	Alive at 30 months	(40)
40	Xiaoyun Xu	2016	10	F	Orbital	2.0	None	N/R	SR + ACT	Alive at 12 months	(41)
41	Hakeem	2020	5	F	Parapharyngeal	5.2	None	N/R	SR	Alive at 36 months	(42)
42	Wang	2020	9	F	Orbital	3.5	Head	N	SR + RT (first treatment) SR + RT (second treatment)	Died at 11 years after head metastasis	(43)
43	Wang	2020	12	F	Orbital	2.4	None	Y	SR (first treatment) SR + RT (second treatment)	Recurrence after 2 months	(43)
44	Wang	2020	1	M	Orbital	3.8	None	Y	SR (first treatment) SR + RT (second treatment)	Recurrence after 8 years	(43)
45	Alkatan	2010	1.25	M	Orbital	2.9	None	N/R	N/R	N/R	(44)
46	Alkatan	2010	6	F	Orbital	N/R	None	N/R	N/R	N/R	(44)
47	Hei	2017	2	F	Orbital	5.0	None	N	SR + RT	Recurrence after 2 months	(45)
48	Hei	2017	6	M	Orbital	2.0	None	Y	SR	Alive at 61 months	(45)
49	Hei	2017	2	M	Orbital	2.0	None	Y	SR + RT	Alive at 49 months	(45)
50	Hei	2017	9	F	Orbital	4.5	None	Y	SR + RT + ACT	Alive at 13 months	(45)
51	Hei	2017	10	F	Orbital	4.5	None	Y	SR	Alive at 13 months	(45)

M, male; F, Female; Y, yes; N, no; N/R, not reported; SR, surgical resection; ACT, adjuvant chemotherapy; RT, radiotherapy; ASPS, alveolar soft part sarcoma.

## Case reports

### Case 1

**Figure 1:**
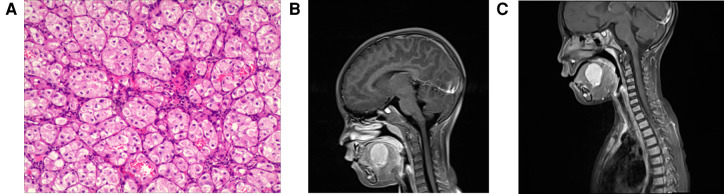
Pathological and radiological examination of case 1. (**A**) Microscopic examination shows that the tumor cells have abundant cytoplasm, eosinophilic, acinar, and nest-like growth. (**B**) MRI of the neck shows that the oval long T1 and long T2 signal masses could be seen on the left side of the mouth bottom, and the maximum cross-section was about 33 mm × 24 mm × 32 mm. (**C**) MRI of necks shows that mass did not shrink significantly after treatment.

In July 2021, the parents of a 5-year-old girl discovered a “pigeon egg” size tumor at the bottom left of her mouth by chance. The patient's medical history was unremarkable. Clinical examination revealed a firm, raised, well-circumscribed, nontender purple mass located in the left dorsal side of the tongue, and several soybean-sized lymph nodes could be touched in the neck and armpit. An excisional biopsy was performed under general anesthesia on 9 September 2021. During histopathologic examination at magnification (Figure 1A), the tumor cells showed abundant cytoplasm, eosinophilic, acinar, and nest-like growth. Immunohistochemistry: CK (−), Vim (+), Desmin (focus +), MyoD1 (−), p63 (partial +), CK5/6 (−), CK8/18 (−), CgA (−), CD56 (−), Syn (−), S100 (−), TFE3 (+), CD34 (vessels +), Ki67 (5% +). Gene detection revealed breakage and fusion occurred between the TFE3 gene and the ASPSCR1 gene. Tumor cell immunophenotype and gene detection support Alveolar soft part sarcoma. There were no obvious abnormalities in the bone marrow examination, cerebrospinal fluid, heart color ultrasound, CT of the lung, abdominal color ultrasound and MRI of the head. The MRI of the neck showed (Figure 1B) that the oval long T1 and long T2 signal masses could be seen on the left side of the mouth bottom, and the maximum cross-section was about 3.3 cm × 2.4 cm × 3.2 cm. The boundary with the surrounding tissue was well-circumscribed, and the enhanced lesions were enhanced and showed high signal intensity on DWI. Multiple small-enlarged lymph nodes were seen in regions I, II, and V of the bilateral neck, and the large ones were located in area II on the left, with a slightly high signal on DWI with a short diameter of about 11 mm. Systemic PET-CT showed that there was no abnormal increase in glucose metabolism in the left mouth, which was consistent with acinar soft tissue sarcoma. Because the tumor was located at the root of the tongue, the operation required total resection of the tongue, which the patient's parents refused. Instead the patient received VDC chemotherapy regimen [Vincristine (VCR) 1.5 mg/m^2^,d1, d8, d15;Pirarubicin (THP) 30 mg/(m^2^*d),d1–d2;Cyclophosphamide (CTX) 1.2 g/m^2^,d1] and IE chemotherapy regimen [Ifosfamide (IFO) 1.8 g/(m^2^*d), d1–d5; Etoposide (VP16) 100 mg/(m^2^*d), d1–d5] and Sunitinib 15 mg/m^2^ for four consecutive weeks, but the mass did not shrink significantly (Figure 1C). Then the patient received I^131^ particle implantation, then followed up after 5 months to reexamine the size of the mass as 25 mm × 19 mm × 27 mm.

### Case 2

**Figure 2:**
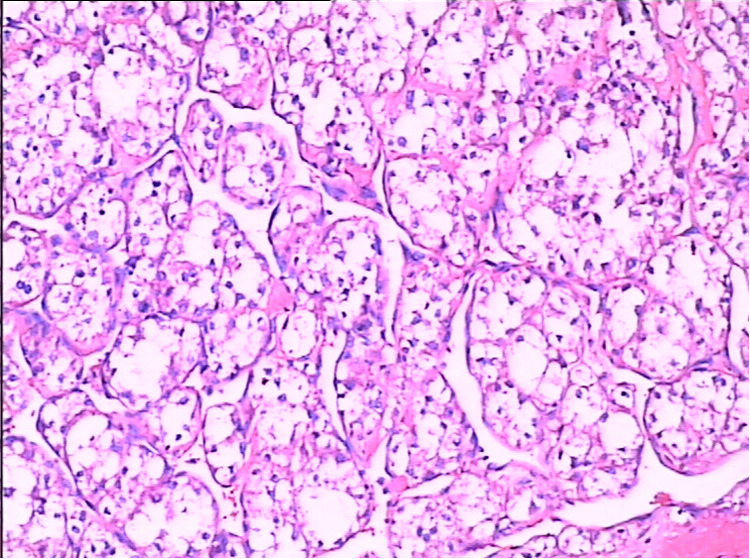
The microscopic examination of case 2 shows that the tumor was acinar-like and abundant in the cytoplasm.

During the physical examination of a 17-year-old female, a mass in the left posterior chest wall was discovered, and CT suggested that the nature of the space-occupying lesion in the left upper chest wall remained unknown. A resection of the left upper posterior chest wall mass and left lower lung nodule was performed on 2 September 2009. The mass was located in the 4th–5th intercostal space of the left posterior chest wall, and the size of the mass was about 4 cm × 5 cm × 5 cm, white, with a rich blood supply, granulation protruded on the surface of the mass, the pedicle extended into the intercostal space, and two 1 cm × 1 cm × 1 cm-sized nodules were palpable around the left lower lobe. Postoperative pathology: (left chest wall) malignant tumor, tumor cells showed acinar arrangement, rich cytoplasm, transparent or eosinophilic, vascular rich. Immunohistochemical: CT (−), HMB45 (−), CEA (−), CD68 (+ −), Syn (−), CD34 (−), CgA (−), F8 (−), TG (−), 5HT (−), GFAP (−), CD1a (−), Ki-67 (−), HHF35 (−), Lys (−), CD10 (−). CK (−), EMA (−), HPC (−), NSE (−), Vim (−), S100 (−), NF (−), and alveolar soft tissue sarcoma was considered. (Left lower lung nodule) two, tumor acinar, rich cytoplasm, red staining or transparent, combined with chest wall mass section immunohistochemical results, consistent with metastatic alveolar soft part sarcoma. She recovered after the operation and was discharged from the hospital on 9 September 2009. The discharge diagnosis was alveolar soft part sarcoma of the left upper posterior chest wall with multiple metastases in both lungs. The patient began to receive four cycles of postoperative IEP chemotherapy [Ifosfamide (IFO) 1.5 g/(m^2^*d) d1–d3, Cisplatin (DDP) 30 mg/(m^2^*d) d1–d3, Epirubicin (E-DAM) 70 mg/(m^2^*d), d1] from 28 September 2009. After four cycles of IEP chemotherapy, CT revealed that the nodules in the lower lobe of both lungs were smaller than before but did not achieve CR. In April 2013, the patient presented again with left chest and back pain, paroxysmal, progressive aggravation of more than one month, accompanied by slight chest tightness, shortness of breath, slight cough, less phlegm, and no fever. CT showed right supraclavicular lymph node metastasis. On September 10 of the same year, a biopsy of the chest and back mass (Figure 2) showed that the tumor showed an acinar-like arrangement with abundant cytoplasm. Immunohistochemistry: Ki-67 (<10% +), CK5/6 (−), CK7 (−), SP-A (−), TTF-1 (−), Vimentin (+), CEA (−), SMA (−), S100 weak (+), CD34 (−). Considering acinar soft tissue sarcoma (recurrence) with bilateral lung and right supraclavicular lymph node metastasis, there are indications for chemotherapy, no chemotherapy taboos, not suitable for surgery and radiotherapy, considering that the patient's previous IEP regimen is effective, the original regimen will still be effective; thus, the patient received IE regimen [Ifosfamide (IFO) 1.5 g/(m^2^*d), d1–d4; Epirubicin (E-DAM) 70 mg/(m^2^*d),d1] for three cycles, then lost follow-up.

### Case 3

**Figure 3:**
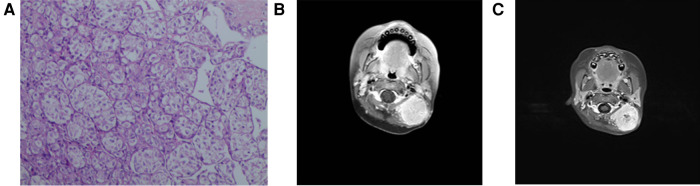
Pathological and radiological examination of case 3. (**A**) Microscopic examination shows mesenchymal malignant tumor, tumor cell cytoplasm transparent or red staining, and some showed acinar arrangement. (**B**) MRI shows a 3.5 cm × 3.0 cm mass and its location. (**C**) MRI shows that the mass was enlarged with a size of about 3.8 cm × 3.3 cm, and there was no enhancement area in the center of the lesion.

A 2-year-old girl was admitted to the hospital on 10 June 2018 due to “finding a mass in the neck for more than 4 months.” Clinical examination revealed a 5.0 cm × 5.5 cm × 2.0 cm mass on the left ear, nontender, no ulceration, poor mobility, several enlarged lymph nodes in the rest of the neck, nontender, well mobility, and the rest of the superficial lymph nodes were not palpable. After admission, a CT of the lung revealed that the lingual segment nodules of the left lower lung were considered to be inflammatory nodules. In order to confirm the diagnosis, a biopsy was performed on the left neck under general anesthesia on 13 June 2018. Three masses of about 2 mm × 2 mm × 2 mm were removed. The biopsy results (Figure 3A) revealed a mesenchymal malignant tumor, tumor cell cytoplasm transparent or red staining, some showed acinar arrangement, tumor cell immunophenotype: CK (−), Vim (+), CD34 (+), MyoD1 (plasma +), SMA (focal +), CD56 (−), CgA (−). S100 (−), Syn (−), Myogenin (−), Desmin (−), TFE3 (+), HMB45 (−), and Ki-67 (about 7% +) supported the diagnosis of alveolar soft part sarcoma. On July 7, PET-CT revealed a soft tissue mass with slightly higher glucose metabolism in the left neck, which was consistent with alveolar soft part sarcoma; several small nodules without a significant increase in glucose metabolism in both lungs, considering the possibility of lung metastasis; lymph nodes with slightly increased glucose metabolism in the left groin, not excluding lymph node metastasis; and small lymph nodes with slightly higher glucose metabolism in the bilateral neck (area II–III) were considered inflammatory lymph nodes. On 12 July 2018, the MRI of the neck (Figure 3B) showed that the size of the neck was about 3.5 cm × 3.0 cm, the structure of the left cephalic semispinalis muscle was unclear, the posterior rectus major muscle and the head clamp muscle were obviously flattened, and the boundary with the mass was unclear; the mass reached the inferior edge of the occipital bone, and no obvious abnormal signal was seen in the occipital bone; inward to the outer edge of the left cervical attachment, no obvious abnormal signal was seen in the cervical vertebrae; forward to the posterior margin of the parotid gland, there was fat space between the parotid gland and the upper edge of the C5 vertebral body. The mass showed iso-T1 and long T2 signal intensity, which was inhomogeneous and obviously enhanced on a contrast-enhanced scan. Multiple enlarged lymph nodes were seen in bilateral parapharyngeal space, cervical I and bilateral cervical II, and the larger ones were located in the left cervical II, about 1.6 cm × 0.9 cm. A patchy long T2 signal was seen in the left maxillary sinus. Considering that the child is young, the tumor is highly malignant, the scope of tumor invasion is large, and the effect of simple operation may be poor, VDC chemotherapy regimen [Vincristine (VCR) 1.5 g/(m^2^*d) d1, d8; Pirarubicin (THP) 60 mg/(m^2^*d) d2, d9; Cyclophosphamide (CTX) 0.3 g/m^2^ d2–d4] and IEV chemotherapy regimen [Ifosfamide (IFO) 1.5 g/(m^2^*d) d2–d6; Etoposide (VP16) 100 mg/(m^2^*d) d2–d6; Vincristine (VCR) 1.5 mg/(m^2^*d) d1, d8] were performed in turn from 12 July 2018. On 13 September 2018, an MRI of the neck (Figure 3C) showed that the mass was enlarged with a size of about 3.8 cm × 3.3 cm, and there was no enhancement area in the center of the lesion. The number of multiple enlarged lymph nodes in the neck was less than before. Considering the poor effect of chemotherapy and the possibility of “lung metastasis,” total resection of alveolar soft part sarcoma in the posterior and posterior skull base was performed under general anesthesia on 25 September 2018. No adjuvant therapy was given after the operation, and no recurrence or metastasis was found after telephone follow-up for 3 years.

## Discussion

ASPS usually occurs between 15 and 35 years old, is rare under 5 years old and over 50 years old, and is more common in children over 10 years old. The incidence rate of females is higher than that of males. The ratio of females to males is 2:1, and there is little sex difference in children (42, 46). To the best of our knowledge, 51 cases of children with ASPS have been reported in the previous literature. Among 54 ASPS, 32 were female and 22 were male. The age of onset was from 11 months to 18 years old, 15 cases under 5 years old, and 23 cases over 10 years old. ASPS can occur in all parts of the body, mostly in the deep soft tissues of the thighs and buttocks in adults, and in children, especially in the tongue and orbit (46). The tumors occurred in the head and neck in 40 cases (14 tongue, 13 orbit, 4 intracranial, 2 thyroid, 2 cheek, 2 paranasal sinus, 1 Parapharyngeal, 1neck, 1 glabella), 2 thigh, 3 bladder, left forearm, left shoulder, chest wall, lung, breast, Uterine Cervix, penis, abdominal wall and left Buttocks in one case respectively. Most of the clinical symptoms of ASPS are slow-growing inert soft tissue masses, but fast-growing masses are also reported. Because the cancer tissue is rich in blood vessels, distant metastasis is easy to occur through the blood tract, lung metastasis is the most common, followed by brain, breast, and bone metastasis. Metastatic diseases first occur in the lungs and then in other parts (47, 48). Due to the slow growth of the mass, it is difficult to attract patients’ attention, metastasis has occurred in most patients at the time of diagnosis, but children are generally found relatively early because of the high level of attention of their parents. In this group, the nature of metastatic mass was determined in 10 cases, of which nine cases were first located in the lung, and seven of these nine patients had occurred at the time of diagnosis, which was consistent with the characteristics reported in the literature.

TFE3 is a member of the basic helix-cyclic-leucine zipper (bHLH-Zip) transcription family of microphthalmia-associated transcription factor/transcription factor E (MITF-TFE). Other members of the same family are MITF, TFEB, and TFEC subtypes (49). The gene of ASPS is characterized by an unbalanced translocation between chromosome X and chromosome 17, that is, der (17) t (X;17) (p11;25). This translocation can induce the fusion of the TFE3 gene at the Xp11.2 site and ASPL gene at 17q25 to produce the ASPSCR-TFE3 chimeric gene, stimulate TFE3 activity, and carcinogenic potential, and induce the overexpression of mitotic MET receptor tyrosine kinase to promote angiogenesis. Tumor cells can obtain a lot of nutrition through this effect, which is conducive to value-added invasion (50). Therefore, the detection of TFE3 immunohistochemistry and ASPL-TFE3 fusion gene is important for the diagnosis of ASPS, while the gene detection of TFE3 alone has high sensitivity and low specificity, and can only be used for primary screening or diagnosis of ASPS with obvious pathological features (51). The immunohistochemical results of TFE3 were reported in 31 of the 54 patients in Table 1, of which two cases were negative. According to the literature report (52), the pathology of ASPS in children and adults is basically the same, the mass generally does not have a complete capsule, tends to be unclear, gray-red, fine texture fish-like, some with necrosis and bleeding, the volume of ASPS mass in children is smaller than that in adults. The most characteristic manifestation under a light microscope is that the tumor cell nest has a unique “organ-like” and acinar structure, that is, a uniform organic nest composed of polygonal and eosinophilic tumor cells is separated by fibrous vascular septum and fine capillary-sized vascular channels, the tumor cells in the center of the nest are lack of adhesion and central cell necrosis to form characteristic acinar type. In children, tumor cells can show diffuse flaky growth without an obvious nest-like structure. Tumor cells contain rich, transparent to granular eosinophilic cytoplasm, with moderately pleomorphic nuclei and obvious nucleoli, occasionally as many as five nucleoli in the same cell. Mitotic images are rare. Under the electron microscope, it can be seen that the cells contain rhomboid or rod-shaped crystal-like inclusion bodies, including crystals and different amounts of glycogen and antiamylase granules, the latter two may be the precursors of crystals. Crystals are aggregates of monocarboxylic acid transporter MCT1 and its cellular chaperone CD147, which can be found in 80% of tumors. Almost all of them had vascular infiltration (9, 13, 23).

ASPS is rare in pediatrics, and often misdiagnosed, especially in unusual sites. The diagnosis of ASPS should be combined with MRI, histopathology, and clinical features. Immunohistochemical and imaging methods play an important role in differential diagnosis. Because ASPS is a highly vascular tumor, MRI is often the first choice in imaging examination (18). ASPS showed slightly high signal intensity on T1W1, a few equal and low signal intensity, which may be related to the slow blood flow in the blood vessels of the tumor, mixed high signal intensity on T2W1, which may be related to bleeding, necrosis and scar formation in the tumor tissue. The hemorrhage was a low signal, the scar tissue was an equal signal, the necrotic sac became a high signal, and multiple twisted vascular flow void signals could be seen in and around the tumor, which may be due to the rapid blood flow velocity. Linear hyperintensity was also seen in the tumor, which may be related to the fibrous septum in the tumor. Obvious inhomogeneous continuous enhancement after enhancement is the characteristic MRI manifestation of ASPS, which may be related to the rich blood vessels and blood sinuses in the tumor, necrosis, and no enhancement in the cystic area. The imaging differential diagnosis should be differentiated from arteriovenous malformations, neurogenic myoma of the extremities, alveolar rhabdomyosarcoma and fibrosarcoma: (1) Arteriovenous malformations: arteriovenous malformations have few solid components, low signal intensity on T1W1 and T2W1 sequences, clear feeding arteries, draining veins and malformed vascular masses, and tortuous empty vessels gather in masses, showing the characteristics of “fast in and fast out” after enhancement. (2) Neurogenic myoma of the extremities: it often occurs in the muscle or intermuscular space, and the typical “target sign” can be seen, that is, the change of low signal surrounding high signal in the center of T2W1. (3) Alveolar rhabdomyosarcoma: it is difficult to distinguish with ASPS in imaging. T1W1 shows iso-signal, T2W1 shows high signal or mixed signal, and obvious enhancement after enhancement, which needs to be differentiated by pathology. (4) Fibrosarcoma: T1W1 showed iso-signal, T2W1 showed high signal or mixed signal, which was obviously enhanced after enhancement, but there was no vascular flow empty signal, which was more common in the middle-aged and elderly (52). ASPS needs to be differentiated from many kinds of tumors in histopathology: (1) ASPSCR1-TFE3 translocation of renal cell carcinoma (RCC): this translocation is mostly balanced translocation and can also express TFE3, but it can constantly express renal cell carcinoma markers and CD10, so it is of clinical significance to pay close attention to renal masses. (2) Adrenocortical carcinoma (ACC): immunohistochemical staining was positive for inhibin, MelanA, Calretinin, CK, and Syn, and it may also express TFE3. (3) Metastatic hepatocellular carcinoma (HCC): CK18, CK20, HepPar1, and AFP were positive by immunohistochemistry, and the tumor cells were arranged in acinar shape in some cases. (4) Paraganglioma: it was distributed along the sympathetic chain, but it was rare in the extremities. The “organ-like” structure could be seen under the microscope, and the immunohistochemical staining of CgA, Syn, and Smur100 were positive. (5) Granulosa cell tumors were more common in the middle-aged and elderly, with rich eosinophilic cytoplasm, small round nuclei in the middle, arranged into solid flakes and nests, immunohistochemical Smur100 and NSE positive, fine granule staining in PAS staining cytoplasm, and TFE3 positive in a few cases. (6) Perivascular epithelioid cell tumor (PEComas): immunohistochemical staining was positive for SMA, HMB-45, MelanA, and a little positive for TFE3 protein, and some of the tumor cells were epithelioid. (7) Malignant melanoma: S100, HMB45, and Melan-A were positive by immunohistochemistry. (8) Alveolar rhabdomyosarcoma: immunohistochemical staining was positive for desmin, MyoD1, and Myogenin, but negative for TFE3. The tumor cells were small round primitive mesenchymal cells and immature striated myoblasts, which were arranged in acinar and nest shape, lacked sinus blood vessels, and were often accompanied by t (1;13) (p36; q14) translocation to form PAX7-FKHR fusion (6, 38).

The first choice for the treatment of ASPS is extensive surgical resection to obtain a tumor-free edge. It is recommended to retain a tumor-free area of 1–1.5 cm around the tumor (20). If the cutting edge of the first operation is not clean or suspicious, reoperation is recommended to expand the scope of resection to avoid residual tumors. If the location of the mass is special and it is difficult to remove it completely; postoperative auxiliary treatment can be considered to prevent recurrence and metastasis (5). The existence of lung metastasis is not a surgical contraindication (28). Cervical lymph node metastasis is a rare clinical entity, and preventive cervical lymph node dissection is generally not recommended unless there are palpable lymph nodes (20). For pediatric people, it usually occurs in the head and neck, mostly in the tongue, if the tumor completely replaces the root of the tongue or the tumor is larger, considering the future quality of life of the children, microvascular free tissue reconstruction (17) or preoperative adjuvant therapy to reduce the mass before surgical resection can be considered. The current views on adjuvant therapy are controversial: (1) Adjuvant chemotherapy: at present, there is no clear definition of the benefits of chemotherapy. There is literature showing that chemotherapy can be used in patients with distant metastasis or patients with a high incidence of advanced disease considering micrometastasis (20), but it is not recommended in most cases, suggesting that ASPS is not sensitive to chemotherapy. However, there are still cases that are sensitive to chemotherapy, such as the seventh patient in Table 1 whose preoperative adjuvant chemotherapy reduced the mass by 30% and then successfully resected it (10). The 30 patients (32) received postoperative adjuvant chemotherapy after partial resection, which completely subsided the residual lesions, all of which indicated the sensitivity of the children to chemotherapy. Among the cases we collected, 17 patients still used chemotherapy during treatment, but the results were not good. The insensitivity of adult ASPS patients to chemotherapy has been established, but the sensitivity of children patients to chemotherapy remains to be determined. (2) Auxiliary radiotherapy: radiotherapy is the same as chemotherapy, the clinical effect is not clear, and the role in improving disease-free survival rate is not clear. It may be used in patients with large tumors, positive incisal margins and complex anatomical structures (20). Among 54 ASPS, the second patient (5) treated with adjuvant radiotherapy reduced the tumor by 40%. It creates conditions for surgery, but the role of radiotherapy in children remains to be studied. (3) High-dose interferon: interferon has the effects of antivirus, inhibition of tumor cell proliferation, regulation of immunity, and anti-tumor. Some studies have shown that high-dose interferon may have a certain effect on patients who cannot achieve extensive resection, but it remains to be studied (53). (4) Targeted therapy: abnormal proliferation and metastasis of blood vessels are the two main characteristics of ASPS. ASPS can express many angiogenic-related molecules, such as angiogenic factor receptor 1 (VEGFR1), angiogenic factor receptor 2 (VEGFR2), angiogenic factor receptor 3 (VEGFR3), epidermal growth factor (EGF), Met, Ret, platelet-derived growth factor beta (PDGFB), platelet-derived growth factor receptor beta (PDGFRB), and non-specific immune-related receptors. Targeted drug therapy is aimed at these molecules. Sunitinib is an oral multi-target TKI that selectively acts on angiogenic factor receptor 1 (VEGFR1), angiogenic factor receptor 2 (VEGFR2), angiogenic factor receptor 3 (VEGFR3), fibroblast growth factor receptor 1 (FGFR1), FMS-like tyrosine kinase 3 (FLT3), platelet-derived growth factor receptor (PDGFR), stem cell factor receptor (SCFR), proto-oncogene Ret, and colony-stimulating factor-1 receptor (CSF1R) (51). It has been used in renal cell carcinoma, gastrointestinal stromal tumor, and pancreatic neuroendocrine tumor. The safety of sunitinib for children is not clear. The maximum tolerated dose for patients without cardiac risk factors is 15 mg/m^2^ for four consecutive weeks and rest for 2 weeks (54). Studies have shown that sunitinib has a good effect on children with advanced ASPS and can improve their quality of life (51), but it should be used cautiously in patients with severe skin injury (34). (5) Immune checkpoint inhibitors: immune checkpoint inhibitors can activate the effect of human autoimmune response against tumors and have a more lasting effect. The main targets are cytolytic T lymphocyte-associated antigen-4 (CTLA-4)/CD152, programmed death receptor-1 (PD-1)/CD279 and programmed death receptor-ligand 1 (PD-L1). At present, it is still in the research stage due to the lack of large sample data (55).

The prognosis of ASPS depends on the patient's age, tumor size, and stage at the time of diagnosis. Many literature have reported that the prognosis of children with ASPS is better than that of adults, and the possible reasons are as follows: (1) age is an important factor determining the prognosis, and the younger the child is, the higher the survival rate is (25); (2) compared with other parts of the body, the prognosis of tongue ASPS is relatively better, especially in young children (23). (3) The tumor size in children is small, and a tumor diameter of less than 5 cm makes the prognosis good (38). Among the 54 cases we collected, 39 cases reported mass size, of which only five cases exceeded 5 cm. Localized ASPS had a good prognosis after gross total resection (42). Small masses in children also provided conditions for surgical resection. (4) Children are common in head and neck, the initial symptoms are easy to be detected and earlier than medical treatment, and the metastasis is less during operation. (5) The degree of cell differentiation in children is high, and invasive behaviors such as infiltration and necrosis may occur in adults.

Therefore, ASPS in children has unique characteristics. When a painless soft tissue mass with slow growth in the head and neck is found in adolescent patients, we should be alert to the possibility of ASPS. Early detection, early diagnosis and early treatment are of great significance for the prognosis of children.
